# Acute Normobaric Hypoxia Increases Post-exercise Lipid Oxidation in Healthy Males

**DOI:** 10.3389/fphys.2017.00293

**Published:** 2017-05-17

**Authors:** Liam P. Kelly, Fabien A. Basset

**Affiliations:** ^1^Faculty of Medicine, Memorial University of NewfoundlandSt. John's, NL, Canada; ^2^School of Human Kinetics and Recreation, Memorial University of NewfoundlandSt. John's, NL, Canada

**Keywords:** normobaric hypoxia, submaximal exercise, substrate oxidation, post-exercise recovery, indirect calorimetry

## Abstract

The primary objective of the current study was to determine the effect of moderate normobaric hypoxia exposure during constant load cycling on post-exercise energy metabolism recorded in normoxia. Indirect calorimetry was used to examine whole body substrate oxidation before, during, 40–60 min post, and 22 h after performing 60 min of cycling exercise at two different fractions of inspired oxygen (F_I_O_2_): (i) F_I_O_2_ = 0.2091 (normoxia) and (ii) F_I_O_2_ = 0.15 (hypoxia). Seven active healthy male participants (26 ± 4 years of age) completed both experimental trials in randomized order with a 7-day washout period to avoid carryover effects between conditions. Resting energy expenditure was initially elevated following cycling exercise in normoxia and hypoxia (Δ 0.14 ± 0.05, kcal min^−1^, *p* = 0.037; Δ 0.19 ± 0.03 kcal min^−1^, *p* < 0.001, respectively), but returned to baseline levels the next morning in both conditions. Although, the same absolute workload was used in both environmental conditions (157 ± 10 W), a shift in resting substrate oxidation occurred after exercise performed in hypoxia while post-exercise measurements were similar to baseline after cycling exercise in normoxia. The additional metabolic stress of hypoxia exposure was sufficient to increase the rate of lipid oxidation (Δ 42 ± 11 mg min^−1^, *p* = 0.019) and tended to suppress carbohydrate oxidation (Δ −55 ± 26 mg min^−1^, *p* = 0.076) 40–60 min post-exercise. This shift in substrate oxidation persisted the next morning, where lipid oxidation remained elevated (Δ 9 ± 3 mg min^−1^, *p* = 0.0357) and carbohydrate oxidation was suppressed (Δ −22 ± 6 mg min^−1^, *p* = 0.019). In conclusion, prior exercise performed under moderate normobaric hypoxia alters post-exercise energy metabolism. This is an important consideration when evaluating the metabolic consequences of hypoxia exposure during prolonged exercise, and future studies should evaluate its role in the beneficial effects of intermittent hypoxia training observed in persons with obesity and insulin resistance.

## Introduction

HYPOXIA is characteristic of exposure to natural and simulated altitude (hypobaric and normobaric) and, for this reason, these terms are used interchangeably to describe a reduction in the partial pressure of inspired oxygen. Although, physiological and acclimatory differences exist between models of environmental hypoxia (Fulco et al., 2013; Coppel et al., 2015), similar reductions in blood oxygen saturation (S*p*O_2_) are observed between methods (Mazzeo, 2008; Saunders et al., 2009). Upon exposure to moderate and greater levels of altitude (>2,000 m) S*p*O_2_ is reduced, which is further affected during exercise due to increased pulmonary blood flow that limits gas exchange at the alveoli (Clark et al., 2007). To maintain perfusion of O_2_ during hypoxic exercise, breathing frequency (Bf), minute ventilation ($\dot{\text{V}}$E), and heart rate (HR) are all elevated above similar workloads performed at sea-level (Mazzeo, 2008). However, these physiological adjustments are limited as maximal oxygen uptake ($\dot{\text{V}}$O_2max_) decreases at a rate of approximately 7% for each 1,000-m increase in elevation above sea-level (Fulco et al., 1998; Peronnet et al., 2006; Mazzeo, 2008; Saunders et al., 2009). During constant-load exercise, both the absolute rate of carbohydrate oxidation (CHOox) and its relative contribution to the fuel mixture are elevated in hypoxia compared to normoxia (Wagner et al., 1986; Sutton et al., 1988; Lundby and Van Hall, 2002; Peronnet et al., 2006). Such a shift in substrate oxidation helps to maintain energy supply in reduced O_2_ environments due to the greater energy yield per liter of O_2_ consumed when glucose is completely oxidized compared to fatty acids and amino acids (Hochachka, 1988; Brooks et al., 1991b; Mazzeo, 2008). This shift in fuel selection may also reflect a change in the relative exercise intensity given that performing the same absolute workload under both environmental conditions contributes to a larger fraction of $\dot{\text{V}}$O_2max_ under hypoxia (Fulco et al., 1998; Saunders et al., 2009). Indeed, relative substrate contributions are similar when workloads are matched for relative exercise intensity under normoxia and hypoxia (Bouissou et al., 1987; Lundby and Van Hall, 2002), which has led to the conclusion that a change in relative exercise intensity rather than hypoxia exposure itself causes the observed effect on substrate partitioning (Lundby and Van Hall, 2002). However, an increased reliance on CHOox in hypoxia has also been observed (Friedmann et al., 2004; Peronnet et al., 2006; Katayama et al., 2010) even when workloads are matched for relative exercise intensity under both environmental conditions. In either case, prolonged exercise performed in hypoxia leads to a greater reliance on endogenous glucose compared to the same absolute and relative workloads completed in normoxia (Peronnet et al., 2006). Therefore, hypoxia exposure during endurance training protocols can be viewed as an additional metabolic stress that the body must overcome to maintain energy supply within exercising muscle.

Although, the cardiopulmonary and metabolic responses to exercise at high altitude are well described (Wagner et al., 1986; Sutton et al., 1988; Brooks et al., 1991b; Benoit et al., 1997; Lundby and Van Hall, 2002; Friedmann et al., 2004; Heubert et al., 2005; Fulco et al., 2011), there is a lack of literature on the post-exercise recovery period. Numerous studies have investigated excessive post-exercise oxygen consumption (EPOC) following exercise performed in normoxia (Gaesser and Brooks, 1984; LaForgia et al., 2006) and its consecutive changes in resting substrate partitioning (Kimber et al., 2003; Kuo et al., 2005; Magkos et al., 2006; Henderson et al., 2007a; Trombold et al., 2013; Henderson and Alderman, 2014). Although, the post-exercise recovery period transiently displays an elevated energy expenditure (EE), a shift in resting substrate partitioning toward lipid energy sources may be the greatest influence of prior exercise on energy metabolism (Kuo et al., 2005; Henderson et al., 2007a; Henderson and Alderman, 2014). Accordingly, elevated rates of resting lipid oxidation (FATox) above time-matched resting controls persist up to 24 h after a single bout of exercise (Henderson et al., 2007a; Henderson and Alderman, 2014). Such increments in post-exercise FATox are associated with the energy expenditure of prior exercise (EEE) (Henderson and Alderman, 2014), the energy deficit (Melanson et al., 2009a) and the extent to which muscle glycogen depletion occurs (Kimber et al., 2003; Trombold et al., 2013). Given the preferential use of endogenous CHO during exercise under hypoxia (Peronnet et al., 2006), it is hypothesized that exercise performed in normobaric hypoxia would lead to an elevated resting FATox post-exposure. However, Katayama et al. (2010) have reported opposing data to this view, displaying an elevated respiratory exchange ratio (RER; $\dot{\text{V}}$CO_2_/$\dot{\text{V}}$O_2_) during the 60 min recovery period from submaximal exercise performed under hypoxia compared to normoxia. These findings might stem from the fact that the recovery period was recorded in hypoxia and only immediately post-exercise. Accordingly, acute passive normobaric hypoxia exposure is sufficient to shift resting energy metabolism toward FATox in overweight men (Workman and Basset, 2012). In addition, there appears to be a synergistic effect of endurance training when combined with normobaric hypoxia as intermittent hypoxia training (IHT) protocols elicit greater improvements in metabolic risk markers at lower mechanical workloads compared to exercise performed in normoxia (Haufe et al., 2008). The efficacy of IHT toward improving insulin sensitivity and decreasing fat mass has been demonstrated in individuals with type 2 diabetes (Mackenzie et al., 2011, 2012) and apparently healthy but overweight individuals (Netzer et al., 2008; Lippl et al., 2010; Wiesner et al., 2010). These reports also align with epidemiological data that demonstrates an inverse association between elevation (<300 vs. ≥3,000 m above sea-level) and obesity prevalence after adjusting for known confounders (Voss et al., 2013). However, the acute effects of prior exercise performed under normobaric hypoxia on post-exercise energy metabolism are currently unknown.

Therefore, the purpose of the current study was to examine the effects of normobaric hypoxia exposure during constant workload exercise (H-CWE) on whole body energy metabolism during the post-exercise recovery period recorded in typical sea-level conditions. It was hypothesized that (a) an increased contribution from carbohydrate energy sources would be observed during H-CWE compared to performing the same constant workload exercise in normoxia (N-CWE) and that (b) the increased reliance on carbohydrate energy sources during H-CWE would alter post-exercise energy metabolism, resulting in an elevated resting EE and a shift in fuel selection toward FATox and away from CHOox up to 22 h post-exercise.

## Methods

### Study participants

The current study was approved by the Human Investigation Committee of the Health Research Ethics Authority of Newfoundland and Labrador (reference number: 08.92) and was carried out in accordance with the recommendations of the Tri-Council Policy Statement: Ethical Conduct for Research Involving Humans (2010), with written informed consent from all subjects. Participants were familiarized with experimental procedures before providing written informed consent in compliance with the Declaration of Helsinki. Seven active healthy male participants were recruited from Memorial University of Newfoundland, St. John's campus, and regional community. The Physical Activity Readiness Questionnaire (PAR-Q) was used to determine physical activity level and to screen for a history of cardiovascular, pulmonary, metabolic, and orthopedic conditions. Participants were excluded from the study if they took prescribed medication of any kind, were smokers, or diagnosed as having: respiratory problems, heart disease, hypertension, chronic, or acute illness, anxiety disorders, and drug or alcohol abuse. Anthropometric and physical fitness characteristics of the study participants are reported in Table 1.

**Table 1:** **Participant characteristics**.

**Parameter**	**Score**
Age (year)	26 ± 4
Height (cm)	175.5 ± 4.0
Weight (kg)	77.5 ± 9.7
BMI (kg m^−2^)	25.2 ± 3.1
Maximal heart rate	187 ± 13
$\dot{\text{V}}$O_2max_ (l min^−1^)	4.01 ± 0.31
Peak power (W)	314 ± 26

### Experimental design

**Figure 1:**
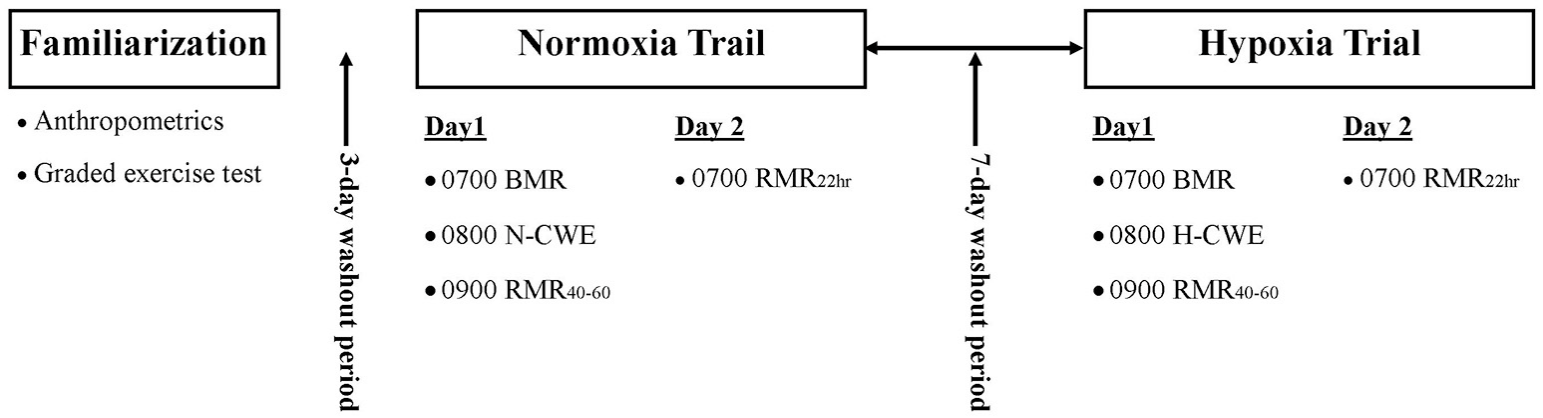
**Experimental design**. Each participant completed a familiarization session followed by two experimental trials in randomized order. Each experimental trial involved a baseline basal metabolic rate (BMR) measurement followed by constant workload exercise performed in normoxia (N-CWE) or in moderate hypoxia (H-CWE) followed by a resting metabolic rate measurements 40–60 min post-exercise (PEMR_40–60_) and again the next morning 22 h into recovery (PEMR_22 h_). All resting measurements were recorded in normoxia.

A crossover study design was used to examine the effect of H-CWE on substrate oxidation during post-exercise recovery recorded in normoxia. As depicted in Figure 1, participants visited the laboratory on five separate occasions over a 3-week period. Participants first completed a maximal graded exercise test (GXT) on the cycle ergometer (describe below) to determine $\dot{\text{V}}$O_2max_ and assign a workload corresponding to 50% of sea-level peak power output (PPO). This sub-maximal workload was used during both N-CWE and H-CWE treatments. Anthropometrics were also collected and participants were familiarized with experimental procedures, including passive exposure to the hypoxic gas mixture (HGM; 15% O_2_) used during H-CWE. The following four visits were split equally between the N-CWE and H-CWE treatments, whose order were randomly assigned with a 7 day washout period between treatments. In order to control for the thermic effect of food and minimize the within-subject variability for substrate partitioning, participants consumed standardized meals (780 kcal; 26 g fat, 98 g carbohydrate, and 28 g protein) in the evening between 17:00 and 18:00 and fasted 12-h prior to each testing day. Each 2 day treatment included a pre-exercise basal metabolic rate (BMR) measurement recorded at 07:00, followed by 60 min of constant workload exercise, a post-exercise resting metabolic rate measurement recorded between 40 and 60 min post-exercise (PEMR_40–60_), and a second post-exercise resting metabolic rate measurement recorded the next morning at 07:00 (PEMR_22 h_). Participants were also requested to maintain a diet log throughout the experimental period and match food intake patterns between treatments. In addition, moderate-to-vigorous intensity and long duration exercise was avoided 48 h prior to each testing session.

### Environmental conditions

A fraction of inspired oxygen (F_I_O_2_) of 15%, corresponding to simulated moderate altitude of approximately 2,750 meters above sea-level, was used during H-CWE while all other measurements were recorded under typical_sea-level_ conditions (F_I_O_2_ = 0.2093). The hypoxic gas mixture was supplied using a generator equipped with a semipermeable filtration membrane (GO2Altitude, Biomedtech Melbourne Australia) that continuously pumped air (120 L min^−1^) into five 120-L Douglas bags. Gas concentration within the Douglas bags was continuously monitored using an oxygen sensor (Rapidox O_2_, Sensotec, Cambridge, UK), ensuring that the target F_I_O_2_ was maintained at 15 ± 0.2%. Participants were interfaced with the hypoxicator using a two-way non-rebreathing valve (2700, Hans Rudolph, Kansas, USA) and tubing. The same interface was used during N-CWE except the tubing was left open to air from a well-ventilated room located near sea level (~300 m). Temperature, barometric pressure, and humidity were also monitored over the course of the study.

### Exercise protocols

#### Peak $\dot{\text{V}}$O_2_ and PPO determination

Participants first completed a maximal incremental exercise test on a magnetic break cycle ergometer (Velotron, Racer Mate, Seattle Washington, USA) to determine $\dot{\text{V}}$O_2max_ and PPO. A ramp protocol was implemented using self-selected cadence above 60 revolutions per minute (RPM) starting at 50 watts and increased by 1 watt every 3 s until participants could no longer maintain the minimum 60 RPM. After 5 min of recovery, a verification phase was implemented to confirm that a true $\dot{\text{V}}$O_2max_ was achieved, as described elsewhere (Rossiter et al., 2006). Briefly, workload was set at 105% of the PPO achieved during the ramp test and participants were asked to maintain this workload for as long as possible. Maximal oxygen uptake was determined when $\dot{\text{V}}$O_2_ during the verification phase did not exceed values recorded during the ramp test. Peak power output was described as the minimum workload required to reach $\dot{\text{V}}$O_max_.

#### Constant workload cycling

The same absolute workload was maintained during N-CWE and H-CWE to match both environmental conditions for the energy expenditure of exercise (EEE). The workload was set at 50% PPO (157±5 W), and the cadence was matched between conditions. Oxygen uptake, carbon dioxide production ($\dot{\text{V}}$CO_2_), breathing frequency (Bf), tidal volume (Vt), and minute ventilation ($\dot{\text{V}}$E) were continuously recorded during both the incremental and constant workload cycling protocols through real-time breath-by-breath sampling using an automated respirometry system (Oxycon Pro, Jaeger, Hochberg, Germany). Participants expired air was collected using a mouthpiece (Reusable Series 9060, Hans Rudolph, Kansas, USA), where the triple-V volume transducer, sample line and housing were connected directly to the mouthpiece. This experimental set-up was chosen during exercise in order to obtain real time respirometry measurements with the capability of quickly removing the mouthpiece for hydration purposes. Blood oxygen saturation and HR were also recorded in line with respirometry data using pulse oximetry (MasimoSET, Masimo Corporation, California, USA). A cutoff value for S*p*O_2_ was set at 80% to control for excessive metabolic stress during H-CWE. In addition, the Lake Louise Acute Mountain Sickness questionnaire was administered at the end of the H-CWE. Participants did not report any symptoms of acute mountain sickness at any time point after the total of 60 min of hypoxia exposure.

### Resting protocols

Resting respirometry measurements were recorded in both treatments before exercise and at two-time points during post-exercise recovery (40–60 min and 22 h) for determination of basal metabolic rate (BMR) and post-exercise metabolic rate (PEMR_40–60_, PEMR_22 h_), respectively. The distinction between BMR and PEMR_22 h_ was made due to the experimental treatments; PEMR_22 h_ cannot be considered as a true BMR because of the carry-over effect of exercise on metabolic response. Therefore, a total of two basal and four recovery resting respirometry measurements were recorded over the study period. The following standardized protocols were followed prior to and during all resting respirometry measurements: (i) participants were instructed not to consume food or energy-containing beverages for 12 h prior to BMR and PEMR_22 h_ but could consume water *ad libitum*, (ii) measurements lasted 45–60 min starting at 07:00, (iii) measurements were recorded in the supine position with participants head supported by a single pillow, (iv) room temperature was maintained at 22°C and lights were dimmed, and (v) participants were instructed to lie motionless but awake and not to talk. Immediately following exercise participants were transferred from the cycle ergometer to the bed where they lay again in the supine position for 60 min while PEMR_40–60_ was recorded. Participants returned to the laboratory the next morning 22 h after N-CWE and H-CWE to record PEMR_22 h_.

The ventilated hood technique was used during all resting measurements to improve accuracy and participant comfort. Flow rate was manipulated to maintain a fraction of expired carbon dioxide (FeCO_2_) between 0.7 and 1.0 within the canopy, as described elsewhere (Simonson and DeFronzo, 1990). Before testing each day, the metabolic cart was given appropriate time to warm-up (≥ 2-h), after which, gas analyzers and volume transducer were calibrated with medically certified calibration gasses (15% O_2_ and 5% CO_2_) and the system built-in calibration functions, according to manufacturer instructions. Also, to ensure accurate performance of the respirometry system, a propane gas verification was performed with a gas mass flow meter set at 200, 300, and 400 ml min^−1^ and measured $\dot{\text{V}}$O_2_ and $\dot{\text{V}}$CO_2_ were compared against theoretical values from stoichiometric calculation.

### Calculations

Measured rates of whole body $\dot{\text{V}}$O_2_ and $\dot{\text{V}}$CO_2_ were used to estimate rates of EE (kcal min^−1^) and disappearance rates (g min^−1^) via oxidation for carbohydrate (CHOox), and lipid (FATox) substrates. Protein oxidation rate (PROox) was estimated at 66 mg min^−1^ based on previously published urinary urea excretion measurements made on 12 h post-absorptive men with normal CHO reserves (Haman et al., 2004). The following formulas were used to estimate CHOox, FATox, and EE during resting and exercise measurements, respectively (Simonson and DeFronzo, 1990; Peronnet and Massicotte, 1991; Jeukendrup and Wallis, 2005).

Resting Stoichiometric Equations:

CHOox = −3.226 $\dot{\text{V}}$O_2_ + 4.585 $\dot{\text{V}}$CO_2_ − 0.461 PROox
FATox = 1.695 $\dot{\text{V}}$O_2_ − 1.701 $\dot{\text{V}}$CO_2_ − 0.319 PROox
EE = 3.87 CHOox + 9.75 FATox + 4.09 PROox

Exercise Stoichiometric Equations:

CHOox = 4.210 $\dot{\text{V}}$CO_2_ − 2.962 $\dot{\text{V}}$O_2_
FATox = 1.695 $\dot{\text{V}}$O_2_ − 1.701 $\dot{\text{V}}$CO_2_
EE = 4.07 CHOox + 9.75 FATox

A negligible contribution from PROox is assumed during exercise.

### Data reduction and statistical analysis

BMR and PEMR_22 h_ measurements were truncated by 25 min out of 45 min data collection in each environmental condition. The procedure discarded the first 15 min and last 10 min to nullify any metabolic rate fluctuation due to familiarization with the ventilated hood and the expected termination of data collection. Respirometry data was then integrated, normalized over time, and used for calculations of substrate oxidation and EE. Data collected during exercise measurements were integrated over time and a mean value representing the entire 60 min exercise period was reported and used for calculations of energy metabolism. The first 40 min of the first post-exercise resting measurements were discarded to remove the influence of CO_2_ retention during recovery due to prior exercise depleting bicarbonate stores (Henderson et al., 2007b), and the final 20 min (40–60 min) were used for calculations. Paired-sample *t*-tests were used to identify differences in metabolic, respirometry, and cardiovascular data between N-CWE and H-CWE. Repeated measures one-way ANOVA was used to test for differences in resting EE and substrate oxidation among BMR, PEMR_40–60_, and PEMR_22 h_ measurements for each experimental condition, followed by post hoc analysis using the Holm-Sidack test when appropriate. Data is reported as means ± SD and statistical significant was set at *p* < 0.05. Statistical Package for the Social Sciences for Mac (version 23.0; Inc., Chicago, IL, USA) and Graph-Pad Prism (Version 7) were used for significance testing and preparing figures.

## Results

### Exercise treatment

In line with previously reported data (Fulco et al., 1998; Heubert et al., 2005; Katayama et al., 2010), exercise performed under moderate hypoxia resulted in significantly lower S*p*O_2_ compared to similar exercise in normoxia (*p* < 0.001). As displayed in Table 2, the decreased F_I_O_2_ during H-CWE lead to a characteristic hypoxic ventilatory response where $\dot{\text{V}}$E (*p* = 0.01) and Bf (*p* = 0.009) were significantly elevated above those recorded during N-CWE, while V_T_ was similar between the two conditions (*p* = 0.56). Although, statistical significance was not reached, HR was also elevated in hypoxia compared to normoxia (Δ 5 ± 2 beat min^−1^; *p* = 0.087). Oxygen uptake and, hence, EEE were significantly lower under hypoxia compared to normoxia (Δ 210 ml min^−1^ and Δ 1.03 kcal min^−1^; *p* = 0.029 and *p* = 0.032, respectively) during cycling exercise performed at a constant absolute workload (157 ± 5 W). This workload corresponded to 69% of $\dot{\text{V}}$O_2max_ under normoxia and approximately 78% of $\dot{\text{V}}$O_2max_ under hypoxia. It therefore appears as though exercise economy ($\dot{\text{V}}$O_2_/W) was improved during H-CWE. However, the diminished $\dot{\text{V}}$O_2_ recorded in hypoxia may actually reflect an increased contribution from non-oxidative energy metabolism (Horscroft and Murray, 2014), which is not reflective of increased exercise economy as these energy reserves are limited (Brooks et al., 1991b; Lundby et al., 2007; Rapoport, 2010). In fact, as displayed in Table 2, FATox decreased by approximately 26% while CHOox increased by only 0.8% during H-CWE compared to N-CWE, which were not significantly different (*p* = 0.163 and 0.906, respectively).

**Table 2:** **Physiological responses to constant workload exercise in normoxia and hypoxia**.

**Parameter**	**Condition**	**Mean**	***S.D***	**95% Confidence interval of difference**		***p*-values**
				**Lower**	**Upper**	
$\dot{\text{V}}$O_2_ (ml min^−1^)	Normoxia	2,759	268	30	396	0.029
	Hypoxia	2,546	159			
$\dot{\text{V}}$CO_2_ (ml min^−1^)	Normoxia	2,481	291	−42	331	0.107
	Hypoxia	2,337	173			
RER	Normoxia	0.898	0.043	−0.058	0.016	0.207
	Hypoxia	0.920	0.046			
S*_*P*_*O_2_ (%)	Normoxia	98.9	1.4	8.7	13.8	<0.001
	Hypoxia	87.7	3.4			
HR (min^−1^)	Normoxia	158.0	16.8	−11.3	1.0	0.087
	Hypoxia	163.1	12.5			
Bf (min^−1^)	Normoxia	33.3	5.0	−10.2	−2.2	0.009
	Hypoxia	39.6	8.2			
Vt (L min^−1^)	Normoxia	2.2	0.4	−0.1	0.1	0.559
	Hypoxia	2.2	0.3			
$\dot{\text{V}}$E (L min^−1^)	Normoxia	70.4	8.3	−20.6	−4.2	0.01
	Hypoxia	82.9	6.7			
CHOox (g min^−1^)	Normoxia	2.274	0.567	−0.464	0.420	0.906
	Hypoxia	2.296	0.498			
FATox (g min^−1^)	Normoxia	0.456	0.181	−0.062	0.292	0.163
	Hypoxia	0.341	0.206			
EEE (kcal min^−1^)	Normoxia	13.70	1.35	0.13	1.94	0.032
	Hypoxia	12.67	0.78			

### Resting protocols

**Figure 2:**
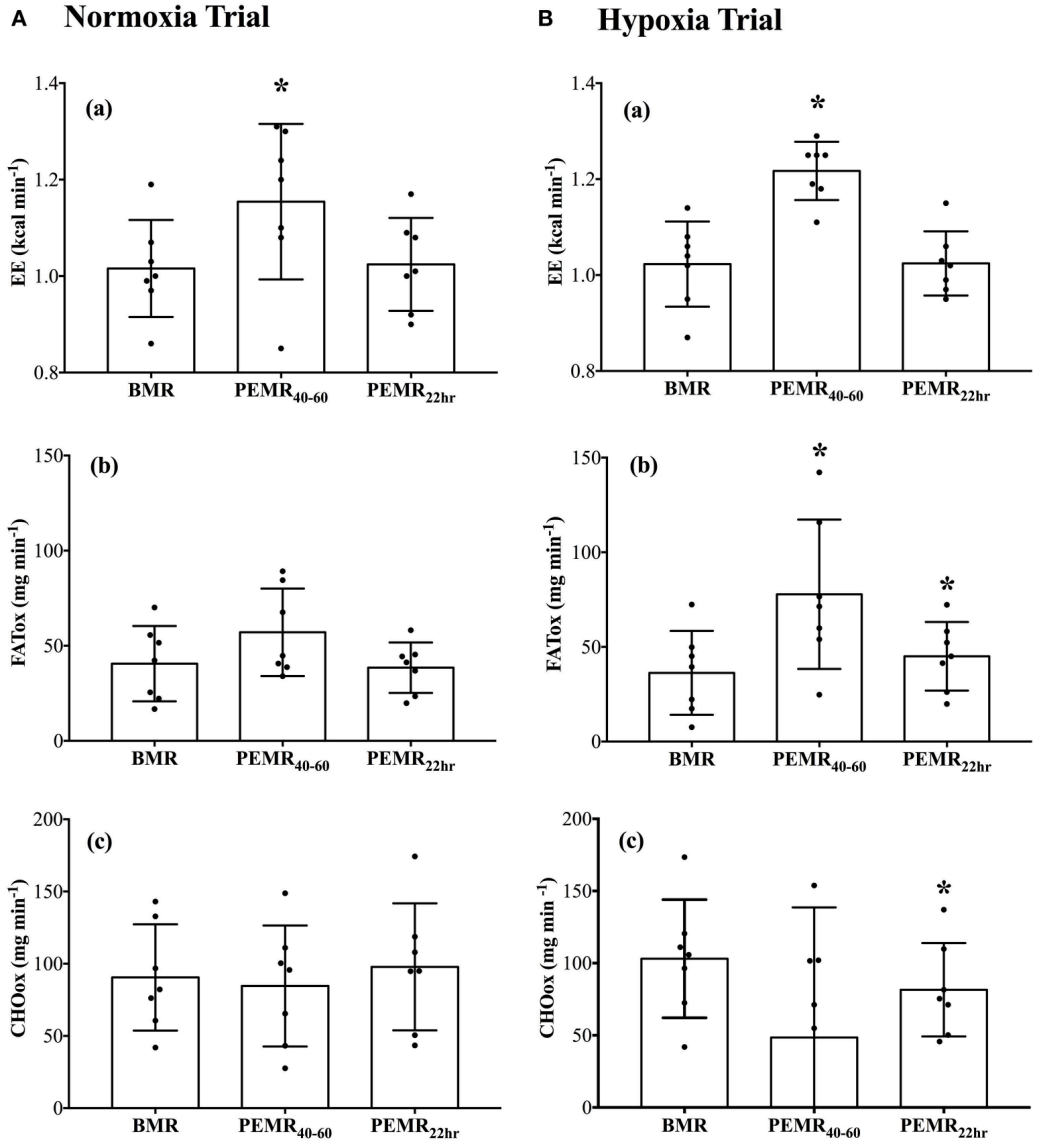
**Resting energy expenditure and substrate oxidation during the normoxia (A)** and hypoxia **(B)** trials. Total rates of (a) energy expenditure (EE; kcal min^−1^), (b) lipid oxidation (FATox; mg min^−1^), and (c) carbohydrate oxidation (CHOox; mg min^−1^) were recorded at baseline (BMR), 40–60 min post exercise (PEMR_40–60_), and the next morning 22-h into recovery (PEMR_22 h_). Significant differences: ^*^p < 0.05 compared with BMR in the respective condition.

BMR measurements recorded before N-CWE and H-CWE were not different from one another on $\dot{\text{V}}$O_2_, $\dot{\text{V}}$CO_2_, HR, EE, FATox, or CHOox. Recall that all measurements in the recovery period were recorded in normoxia. As displayed in Figure 2, EE was elevated above BMR after N-CWE (Δ 0.14 kcal min^−1^; *p* = 0.037) and H-CWE (Δ 0.19 kcal min^−1^; *p* < 0.001) during PEMR_40–60_. However, substrate partitioning was only affected after H-CWE (Figure 2) displaying a higher FATox (Δ 41.5 mg min^−1^; *p* = 0.019) and a trend toward a lower CHOox (Δ −54.6 mg min^−1^; *p* = 0.076). This shift in substrate oxidation resulted in a 24% reduction and a 28% increase in the relative contribution of CHOox (16 vs. 40%) and FATox (62 vs. 34%) to EE during PEMR_40–60_ compared to BMR in the hypoxia condition. A large standard deviation was observed for CHOox during PEMR_40–60_ after H-CWE and two subjects had negative values, which may indicate that the bicarbonate stores were not completely restored in these subjects. Concomitantly, HR remained elevated throughout PEMR_40–60_ after N-CWE (Δ 21 ± 5 bpm; *p* = 0.004) and H-CWE (Δ 23 ± 3 bpm; *p* < 0.001), while S*p*O_2_ returned to baseline values in both conditions. The statistical analysis also revealed a carryover effect the next morning on resting substrate oxidation after H-CWE. In fact, resting FATox remained elevated (Δ 9 ± 3 mg min^−1^; *p* = 0.0357) while CHO oxidation was suppressed (Δ −22 ± 6 mg min^−1^; *p* = 0.019) 22 h after H-CWE, while no effect was observed the next morning after N-CWE (see Supplementary Figure 1). Accordingly, a 9% reduction in CHOox (31 vs. 40%) and an 8% increase in FATox (42 vs. 34%) was maintained at 22 h post-exercise compared to the BMR in the hypoxia trial. Resting EE returned to baseline values the next morning after H-CWE and N-CWE.

## Discussion

The novelty of this study lies in its paradigm—while previous studies have evaluated the effect of hypoxia exposure during both exercise and post-exercise recovery, the current design adds to this literature by evaluating the recovery period under typical sea level conditions. In addition to expanding our understanding of the metabolic consequences of environmental hypoxia exposure, this data may also provide insight into the beneficial effects of normobaric hypoxia training protocols. Intermittent hypoxia training improves metabolic risk markers in healthy obese (Haufe et al., 2008; Netzer et al., 2008; Wiesner et al., 2010) and chronic disease populations (Mackenzie et al., 2011, 2012). During IHT interventions, participants are exposed to environmental hypoxia during the individulal exercise bouts while recovery periods occur in typical sea-level conditions. However, the extent to which the recovery period between individual bouts of exercise in hypoxia contributes to such metabolic acclimations is unclear. The current data demonstrates that the post-exercise recovery period is indeed affected by prior exercise performed in moderate normobaric hypoxia. Although, resting EE was initially elevated after both exercise protocols, resting substrate partitioning was only altered after performing the submaximal exercise in hypoxia. A significant carryover effect was observed the next morning after H-CWE displaying an increased contribution from FATox and a suppressed CHOox while resting EE returned to baseline values in both conditions. The current findings are in contrast to the elevated RER reported by Katayama et al. (2010) during post-exercise recovery in normobaric hypoxia. However, participants in that study remained in hypoxia for both the bout of exercise and throughout the 60 min recovery period, which may explain the discrepancy in these findings.

Similar exercise performed in normoxia was not sufficient to alter post-exercise energy metabolism. Aside from an elevated EE recorded immediately following N-CWE, no significant differences in metabolic parameters were recorded during PEMR_40–60_ or PEMR_22 h_ compared to BMR in the normoxia condition. Using similar workloads to the current study (i.e., 60 min at 60% of $\dot{\text{V}}$O_2max_), Magkos et al. (2007, 2008) also failed to report a significant effect on resting substrate oxidation the morning after an evening bout of exercise in normoxia. However, other original works (Kuo et al., 2005; Henderson et al., 2007a) and a recent meta-analysis (Henderson and Alderman, 2014) have reported conflicting findings. These studies describe elevated rates of resting FATox unto 23 h after performing a single bout of submaximal exercise (60 min at 45–65% of $\dot{\text{V}}$O_2max_) in normoxia. Differences in dietary controls used during the study periods may explain these discrepancies. Kuo et al. (2005) and Henderson et al. (2007a) controlled for 24 h nutrient intake ensuring that the same diet was consumed in exercise and non-exercise trials while Magkos et al. (2007) did not. In fact, Melanson et al. (2002) reported that 24 h FATox was not different between exercise days (30 min at 40 and 70% of $\dot{\text{V}}$O_2max_) and non-exercise days when energy balance was maintained through consumption of additional calories to replace the energy expended during prior exercise. This group reproduced these findings in obese subjects, older adults and endurance trained individuals (Melanson et al., 2009a) and later concluded that exercise has little effect on daily FATox in non-fasted individuals (Melanson et al., 2009b). However, their experimental design is criticized for having a relatively high dietary CHO intake and increasing energy consumption beyond that expended during the actual activity (Henderson and Alderman, 2014). In the current study, subjects performed constant workload cycling in normoxia and hypoxia (i.e., matched for EEE) and stringent diets were not imposed. Therefore, a larger energy deficit imposed during H-CWE cannot explain why performing exercise under hypoxia shifted resting substrate partitioning toward FATox up to 22 h post-exercise while no effect was observed after performing the same workload in typical sea level condition. In addition, to control for the thermic effect of food and minimize the within-subject variability for substrate partitioning, participants consumed the same standardized meals (780 kcal; 26 g fat, 98 g carbohydrate, and 28 g protein) in the evening between 17:00 and 18:00 and fasted 12 h prior to each testing day.

Oxygen uptake and, thus, EEE were lower during H-CWE compared to N-CWE in the current experimental design. Similar reductions in $\dot{\text{V}}$O_2_ during constant load cycling have been reported in hypoxia compared to normoxia (Wagner et al., 1986; Sutton et al., 1988). During steady state exercise at 120 W for 7–10 min at a simulated altitude of approximately 3,000 m above sea level, Wagner et al. (1986) reported that $\dot{\text{V}}$O_2_ was reduced by 150 ml min^−1^ compared to the same workload at sea level. However, other studies have found no significant differences in $\dot{\text{V}}$O_2_ during constant load exercise performed in normoxia and hypoxia (Brooks et al., 1991a,b; Lundby and Van Hall, 2002; Peronnet et al., 2006). It is unclear why these discrepancies exist, however, differences in pre-exercise nutritional controls should be considered. For example, Barnholt et al. (2006) demonstrated that calorie restriction can modulate the endocrine responses to high altitude exposure, which they interpreted as an enhance energy preservation during prolonged calorie restriction. In addition, Charlot et al. (2013) reported increased CHOox and lower levels of O_2_ desaturation during exercise under hypoxia after consuming a high carbohydrate meal compared to energy matched high protein meal. Given that the energy yield per unit of oxygen consumed is greater for CHO than the other substrates (Hochachka, 1988; Roberts et al., 1996; Mazzeo, 2008), an increased contribution from CHO substrates during constant load exercise in hypoxia might at least partially explain the enhanced metabolic economy ($\dot{\text{V}}$O_2_/W). Indeed, a greater dependence on CHO substrates (Brooks et al., 1991b) and a shift away from FATox (Horscroft and Murray, 2014) are common observations of high altitude acclimatization. It is less clear if this shift away from FATox leads to increased metabolic economy at high altitude. Increased metabolic economy has been described in high altitude natives (Hochachka et al., 1991) and in lowlanders after high altitude trekking (Green et al., 2000). However, data collapsed from several studies does not reveal improved metabolic economy after high altitude acclimatization (Lundby et al., 2007). In the current study, rates of whole body CHOox were essentially the same during N-CWE and H-CWE (2.274 vs. 2.296 g min^−1^, respectively) and, although not statistically significant, the main effect on EEE seems to be a reduction in the rate of FATox during H-CWE. It is then plausible that non-oxidative mechanisms provided the additional energy needed to meet the requirements of constant load cycling in normobaric hypoxia. In fact, a recent meta-analysis evaluating the effect of environmental hypoxia on metabolic process in mammalian skeletal muscle suggests that oxidative metabolism and whole body fatty acid metabolism are down regulated during hypoxia exposure (Horscroft and Murray, 2014). In addition, the elevated EE recorded after H-CWE is consistent with an increased contribution from non-oxidative mechanisms during the prior bout of exercise as the O_2_ debt would be repaid during recovery from exercise (Gaesser and Brooks, 1984).

It is not appropriate to draw conclusions on the underlying mechanisms responsible for the observed shift in substrate oxidation after hypoxia exposure as it is beyond the scope of the present study. However, increased utilization of endogenous CHO reserves during H-CWE is certainly a plausible explanation. Although, differences in substrate oxidation between N-CWE and H-CWE were not significantly different, EEE was significantly lower during H-CWE. Given that the cycle ergometer workload was matched between environmental conditions and therefore matched for EEE, non-oxidative mechanisms might have provided the additional energy needed to maintain the workload. For example, assuming that non-oxidative energy sources made up for the 122 kcal energy deficit in hypoxia and that 3 moles of ATP are produced per mole of glycosyl unit from fast glycolysis (Ferrannini, 1988) then approximately 280 g of glycogen would be required. These calculations are hypothetical as changes in glycogen content were not measured in the current study, however, they point to the possibility that hypoxia increased glycogen utilization. Also, hypoxia exposure may cause a shift from exogenous glucose to more endogenous sources of carbohydrate to maintain ATP supply during constant load exercise. Using indirect calorimetry combined with stable isotope tracer techniques, Peronnet et al. (2006) demonstrated that the increased contribution from CHOox observed during exercise in hypoxia was due to an increased reliance on endogenous energy sources. Glycogen-depleting exercise has previously been described to increase resting FATox during the post-exercise recovery period when muscle glycogen replenishment is a priority and plasma, as well as intramuscular triglycerides, are likely to be important fuel sources for oxidative energy production (Kimber et al., 2003). Muscle biopsy and/or carbon tracer techniques were not available to determine muscle glycogen depletion and exogenous vs. endogenous glucose utilization. Therefore, further research is required to elucidate the precise mechanisms.

The current experimental design may help to explain the seemingly paradoxical observation of accelerated weight loss during normobaric hypoxia training at lower total EEE compared to sea-level exercise interventions (Haufe et al., 2008; Netzer et al., 2008; Wiesner et al., 2010). Enhanced lipid metabolism due to intermittent hypoxia exposure has been suggested as a likely explanation for such outcomes (Wiesner et al., 2010; Workman and Basset, 2012). The current findings indicate that the recovery period between the individual bouts of exercise performed in hypoxia may also contribute to the accelerated weight loss during IHT. As described elsewhere (Flatt, 2004, 2012), the amount of glycogen an individual habitually maintains can influence adiposity and perhaps the most significant effect of exercise training on fat mass is to reduce the extent to which the body's glycogen stores are maintained between individual bouts of exercise. From this point of view, exercise strategies that rely heavily on endogenous glucose may be an effective strategy to increase 24 h lipid oxidation and thus induce decreases in body fat mass. This is in contrast to a meta-analysis that evaluated the effect of prior exercise on resting FATox and revealed that the increased FATox observed during post-exercise recovery was related to the energy expended during the prior exercise and the individuals level of fitness while exercise intensity had no effect when matched for EEE (Henderson and Alderman, 2014). However, the range of exercise intensities included in that meta-analysis was relatively narrow, where most studies compared workloads between 45 and 65% of $\dot{\text{V}}$O_2max_ and no studies looked at exercise intensities beyond 75% of $\dot{\text{V}}$O_2max_. In fact, Trombold et al. (2013) demonstrated that high intensity exercise (alternating 2 min at 25% and 2 min at 90% of $\dot{\text{V}}$O_2max_) was more effective than moderate intensity exercise (50% of $\dot{\text{V}}$O_2max_ for 60 min) at increasing postprandial lipid oxidation when matched for EEE, which they suggested was due to contributed to increased muscle glycogen depletion during high intensity exercise. The challenge with implementing such exercise intensities in untrained obese populations is the high risk of injury and such participants are unlike to maintain the required workloads. This is where IHT could prove to be beneficial by increasing the metabolic stress (relative workload) without increasing the mechanical stress. Such exercise strategies may be beneficial for individuals that have metabolic risk factors but are unable to maintain workloads sufficient to achieve cardiovascular/metabolic benefits such as stroke survivors.

Methodological considerations of the current study design should be discussed. Firstly, calculations of substrate oxidation using respirometry measurements are based on the assumption that $\dot{\text{V}}$O_2_ and $\dot{\text{V}}$CO_2_ recorded at the mouth reflect that at the tissue level (Ferrannini, 1988; Simonson and DeFronzo, 1990; Jeukendrup and Wallis, 2005). During intense exercise, hyperventilation increases $\dot{\text{V}}$CO_2_ at the mouth above $\dot{\text{V}}$CO_2_ in tissues, which would result in an inflated RER (Jeukendrup and Wallis, 2005). In the present study, $\dot{\text{V}}$E was significantly elevated under hypoxia compared to normoxia. However, $\dot{\text{V}}$CO_2_ was relatively stable and reached similar values during exercise under both environmental conditions. This indicates that the elevated $\dot{\text{V}}$E recorded in hypoxia did not lead to an inflated RER compared to the normoxia condition. Likewise, CO_2_ retention is transiently increased for the first hour of recovery following high intensity exercise in order to replenish bicarbonate pools (Henderson et al., 2007b), which would be reflected in a low RER and over-estimation of FATox. Hence, the large variability in substrate oxidation during PEMR_40–60_ after H-CWE may partially be explained by CO_2_ retention; however, the observed elevation in resting FATox persisted 22 h post-exercise when bicarbonate stores would have been restored. Therefore, calculations of substrate oxidation used in the current study can be taken to reflect changes occurring at the tissue level. Secondly, stringent diets were not imposed throughout the current study and therefore we cannot exclude the possibility that energy intake and/or macronutrient composition may have been different between the experimental trials. However, participants maintained a diet log throughout the experimental period and matched food intake patterns between experimental trials. Previous studies have imposed strict dietary controls throughout the study period ensuring that a net energy deficit was maintained during the exercise trials (Kuo et al., 2005; Henderson et al., 2007a). It has been argued that such dietary controls lead to increased resting FATox because of the energy deficit on exercise days rather than prior exercise itself (Melanson et al., 2009a). Therefore, keeping participants on their routine food and using a crossover study design seemed the natural choice in order to uninfluence resting substrate partitioning by a special diet.

In conclusion, the results of the current study provide strong evidence that the post-exercise recovery period is affected by hypoxia exposure during the prior bout of exercise. As such, the post-exercise recovery period is an important consideration when evaluating the effect of hypoxia exposure on whole body energy metabolism. This is one among the few studies to record the recovery period under typical sea-level conditions up to 22 h after submaximal exercise performed in moderate normobaric hypoxia. The results indicate that moderate hypoxia exposure during submaximal exercise alters post-exercise whole body energy metabolism, shifting fuel selection toward lipid energy sources. Although, CHOox was similar during exercise in both environmental conditions, it is argued that the decreased EEE observed during H-CWE may reflect an increased contribution from non-oxidative metabolism during hypoxia exposure and that this would lead to a larger depletion of endogenous CHO reserves. Correspondingly, increased muscle glycogen depletion is a possible mechanism to explain the shift toward lipid energy sources after H-CWE. However, it is beyond the scope of the present study to elucidate the underlying mechanics and future studies are required. Moderate normobaric hypoxia exposure was well tolerated during workloads completed in the current study and these findings suggest that it provided a superior metabolic stress over traditional exercise toward increasing post-exercise FATox. However, caution should be exercised when interpreting these results given the small sample size and study population, and therefore, further work is needed before they can be applied to female, older, and unhealthy populations.

## Author contributions

LK: designed the experiment, performed calculations, interpreted findings, and wrote the manuscript. FB: conceived of and designed the experiment, performed calculations, analyzed results, and interpreted findings.

### Conflict of interest statement

The authors declare that the research was conducted in the absence of any commercial or financial relationships that could be construed as a potential conflict of interest.

## Supplementary material

Supplementary Figure 1 **Standardized mean difference effect size (Cohen's d)**. Magnitude of difference (effect size ± confidence limit) in carbohydrate (CHOox) and lipid (FATox) oxidation 22 h after constant-workload cycling exercise in the normoxia **(A)** and hypoxia **(B)** trials compared to baseline BMR in the respective condition.

## References

[B1] Barnholt K. E. Hoffman A. R. Rock P. B. Muza S. R. Fulco C. S. Braun B.. (2006). Endocrine responses to acute and chronic high-altitude exposure (4,300 meters): modulating effects of caloric restriction. Am. J. Physiol. Endocrinol. Metab. 290, E1078–E1088. 10.1152/ajpendo.00449.2005 16380390

[B2] Benoit H. Busso T. Prieur F. Castells J. Freyssenet D. Lacour J. R.. (1997). Oxygen uptake during submaximal incremental and constant work load exercises in hypoxia. Int. J. Sports Med. 18, 101–105. 10.1055/s-2007-972603 9081265

[B3] Bouissou P. Guezennec C. Y. Defer G. Pesquies P. (1987). Oxygen consumption, lactate accumulation, and sympathetic response during prolonged exercise under hypoxia. Int. J. Sports Med. 8, 266–269. 10.1055/s-2008-1025667 3667023

[B4] Brooks G. A. Butterfield G. E. Wolfe R. R. Groves B. M. Mazzeo R. S. Sutton J. R.. (1991a). Decreased reliance on lactate during exercise after acclimatization to 4,300 m. J. Appl. Physiol. (1985). 71, 333–341. 1917759 10.1152/jappl.1991.71.1.333

[B5] Brooks G. A. Butterfield G. E. Wolfe R. R. Groves B. M. Mazzeo R. S. Sutton J. R.. (1991b). Increased dependence on blood glucose after acclimatization to 4,300 m. J. Appl. Physiol. (1985). 70, 919–927. 2022585 10.1152/jappl.1991.70.2.919

[B6] Charlot K. Pichon A. Richalet J. P. Chapelot D. (2013). Effects of a high-carbohydrate versus high-protein meal on acute responses to hypoxia at rest and exercise. Eur. J. Appl. Physiol. 113, 691–702. 10.1007/s00421-012-2472-z 22918557

[B7] Clark S. A. Bourdon P. C. Schmidt W. Singh B. Cable G. Onus K. J.. (2007). The effect of acute simulated moderate altitude on power, performance and pacing strategies in well-trained cyclists. Eur. J. Appl. Physiol. 102, 45–55. 10.1007/s00421-007-0554-0 17882451

[B8] Coppel J. Hennis P. Gilbert-Kawai E. Grocott M. P. (2015). The physiological effects of hypobaric hypoxia versus normobaric hypoxia: a systematic review of crossover trials. Extrem. Physiol. Med. 4:2. 10.1186/s13728-014-0021-6 25722851 PMC4342204

[B9] Ferrannini E. (1988). The theoretical bases of indirect calorimetry: a review. Metab. Clin. Exp. 37, 287–301. 10.1016/0026-0495(88)90110-2 3278194

[B10] Flatt J. P. (2004). Carbohydrate-fat interactions and obesity examined by a two-compartment computer model. Obes. Res. 12, 2013–2022. 10.1038/oby.2004.252 15687403

[B11] Flatt J. P. (2012). Misconceptions in body weight regulation: implications for the obesity pandemic. Crit. Rev. Clin. Lab. Sci. 49, 150–165. 10.3109/10408363.2012.712904 22913406

[B12] Friedmann B. Bauer T. Menold E. Bartsch P. (2004). Exercise with the intensity of the individual anaerobic threshold in acute hypoxia. Med. Sci. Sports Exerc. 36, 1737–1742. 10.1249/01.MSS.0000142307.62181.37 15595295

[B13] Fulco C. S. Beidleman B. A. Muza S. R. (2013). Effectiveness of preacclimatization strategies for high-altitude exposure. Exerc. Sport Sci. Rev. 41, 55–63. 10.1097/JES.0b013e31825eaa33 22653279

[B14] Fulco C. S. Muza S. R. Beidleman B. A. Demes R. Staab J. E. Jones J. E.. (2011). Effect of repeated normobaric hypoxia exposures during sleep on acute mountain sickness, exercise performance, and sleep during exposure to terrestrial altitude. Am. J. Physiol. Regul. Integr. Comp. Physiol. 300, R428–R436. 10.1152/ajpregu.00633.2010 21123763

[B15] Fulco C. S. Rock P. B. Cymerman A. (1998). Maximal and submaximal exercise performance at altitude. Aviat. Space Environ. Med. 69, 793–801. 9715971

[B16] Gaesser G. A. Brooks G. A. (1984). Metabolic bases of excess post-exercise oxygen consumption: a review. Med. Sci. Sports Exerc. 16, 29–43. 10.1249/00005768-198401000-00008 6369064

[B17] Green H. J. Roy B. Grant S. Hughson R. Burnett M. Otto C. (2000). Increases in submaximal cycling efficiency mediated by altitude acclimatization. J. Appl. Physiol. (1985). 89, 1189–1197. 10956368 10.1152/jappl.2000.89.3.1189

[B18] Haman F. Legault S. R. Weber J. M. (2004). Fuel selection during intense shivering in humans: EMG pattern reflects carbohydrate oxidation. J. Physiol. 556(Pt 1), 305–313. 10.1113/jphysiol.2003.055152 14742724 PMC1664890

[B19] Haufe S. Wiesner S. Engeli S. Luft F. C. Jordan J. (2008). Influences of normobaric hypoxia training on metabolic risk markers in human subjects. Med. Sci. Sports Exerc. 40, 1939–1944. 10.1249/MSS.0b013e31817f1988 18845972

[B20] Henderson G. C. Alderman B. L. (2014). Determinants of resting lipid oxidation in response to a prior bout of endurance exercise. J. Appl. Physiol. (1985). 116, 95–103. 10.1152/japplphysiol.00956.2013 24235102

[B21] Henderson G. C. Fattor J. A. Horning M. A. Faghihnia N. Johnson M. L. Mau T. L. (2007a). Lipolysis and fatty acid metabolism in men and women during the postexercise recovery period. J. Physiol. 584(Pt 3), 963–981. 10.1113/jphysiol.2007.137331 17855762 PMC2277001

[B22] Henderson G. C. Fattor J. A. Horning M. A. Faghihnia N. Luke-Zeitoun M. Brooks G. A. (2007b). Retention of intravenously infused [^13^C]bicarbonate is transiently increased during recovery from hard exercise. J. Appl. Physiol. (1985). 103, 1604–1612. 10.1152/japplphysiol.00309.2007 17702837

[B23] Heubert R. A. Quaresima V. Laffite L. P. Koralsztein J. P. Billat V. L. (2005). Acute moderate hypoxia affects the oxygen desaturation and the performance but not the oxygen uptake response. Int. J. Sports Med. 26, 542–551. 10.1055/s-2004-821329 16195987

[B24] Hochachka P. W. (1988). Patterns of O_2_-dependence of metabolism. Adv. Exp. Med. Biol. 222, 143–151. 10.1007/978-1-4615-9510-6_16 3364235

[B25] Hochachka P. W. Stanley C. Matheson G. O. McKenzie D. C. Allen P. S. Parkhouse W. S. (1991). Metabolic and work efficiencies during exercise in Andean natives. J. Appl. Physiol. (1985). 70, 1720–1730. 2055851 10.1152/jappl.1991.70.4.1720

[B26] Horscroft J. A. Murray A. J. (2014). Skeletal muscle energy metabolism in environmental hypoxia: climbing towards consensus. Extrem. Physiol. Med. 3:19. 10.1186/2046-7648-3-19 25473486 PMC4253994

[B27] Jeukendrup A. E. Wallis G. A. (2005). Measurement of substrate oxidation during exercise by means of gas exchange measurements. Int. J. Sports Med. 26(Suppl. 1), S28–S37. 10.1055/s-2004-830512 15702454

[B28] Katayama K. Goto K. Ishida K. Ogita F. (2010). Substrate utilization during exercise and recovery at moderate altitude. Metab. Clin. Exp. 59, 959–966. 10.1016/j.metabol.2009.10.017 20036404

[B29] Kimber N. E. Heigenhauser G. J. Spriet L. L. Dyck D. J. (2003). Skeletal muscle fat and carbohydrate metabolism during recovery from glycogen-depleting exercise in humans. J. Physiol. 548(Pt 3), 919–927. 10.1113/jphysiol.2002.031179 12651914 PMC2342904

[B30] Kuo C. C. Fattor J. A. Henderson G. C. Brooks G. A. (2005). Lipid oxidation in fit young adults during postexercise recovery. J. Appl. Physiol. (1985). 99, 349–356. 10.1152/japplphysiol.00997.2004 15591292

[B31] LaForgia J. Withers R. T. Gore C. J. (2006). Effects of exercise intensity and duration on the excess post-exercise oxygen consumption. J. Sports Sci. 24, 1247–1264. 10.1080/02640410600552064 17101527

[B32] Lippl F. J. Neubauer S. Schipfer S. Lichter N. Tufman A. Otto B.. (2010). Hypobaric hypoxia causes body weight reduction in obese subjects. Obesity (Silver Spring). 18, 675–681. 10.1038/oby.2009.509 20134417

[B33] Lundby C. Calbet J. A. Sander M. van Hall G. Mazzeo R. S. Stray-Gundersen J. (2007). Exercise economy does not change after acclimatization to moderate to very high altitude. Scand. J. Med. Sci. Sports 17, 281–291. 10.1111/j.1600-0838.2006.00530.x 17501869

[B34] Lundby C. Van Hall G. (2002). Substrate utilization in sea level residents during exercise in acute hypoxia and after 4 weeks of acclimatization to 4100 m. Acta Physiol. Scand. 176, 195–201. 10.1046/j.1365-201X.2002.01030.x 12392499

[B35] Mackenzie R. Elliott B. Maxwell N. Brickley G. Watt P. (2012). The effect of hypoxia and work intensity on insulin resistance in type 2 diabetes. J. Clin. Endocrinol. Metab. 97, 155–162. 10.1210/jc.2011-1843 21994967

[B36] Mackenzie R. Maxwell N. Castle P. Brickley G. Watt P. (2011). Acute hypoxia and exercise improve insulin sensitivity (S(I) (2^*^)) in individuals with type 2 diabetes. Diabetes Metab. Res. Rev. 27, 94–101. 10.1002/dmrr.1156 21218513

[B37] Magkos F. Patterson B. W. Mohammed B. S. Mittendorfer B. (2007). A single 1-h bout of evening exercise increases basal FFA flux without affecting VLDL-triglyceride and VLDL-apolipoprotein B-100 kinetics in untrained lean men. Am. J. Physiol. Endocrinol. Metab. 292, E1568–E1574. 10.1152/ajpendo.00636.2006 17264219

[B38] Magkos F. Tsekouras Y. E. Prentzas K. I. Basioukas K. N. Matsama S. G. Yanni A. E.. (2008). Acute exercise-induced changes in basal VLDL-triglyceride kinetics leading to hypotriglyceridemia manifest more readily after resistance than endurance exercise. J. Appl. Physiol. (1985). 105, 1228–1236. 10.1152/japplphysiol.90761.2008 18669933

[B39] Magkos F. Wright D. C. Patterson B. W. Mohammed B. S. Mittendorfer B. (2006). Lipid metabolism response to a single, prolonged bout of endurance exercise in healthy young men. Am. J. Physiol. Endocrinol. Metab. 290, E355–E362. 10.1152/ajpendo.00259.2005 16219668

[B40] Mazzeo R. S. (2008). Physiological responses to exercise at altitude: an update. Sports Med. 38, 1–8. 10.2165/00007256-200838010-00001 18081363

[B41] Melanson E. L. Gozansky W. S. Barry D. W. Maclean P. S. Grunwald G. K. Hill J. O. (2009a). When energy balance is maintained, exercise does not induce negative fat balance in lean sedentary, obese sedentary, or lean endurance-trained individuals. J. Appl. Physiol. (1985). 107, 1847–1856. 10.1152/japplphysiol.00958.2009 19833807 PMC3774345

[B42] Melanson E. L. MacLean P. S. Hill J. O. (2009b). Exercise improves fat metabolism in muscle but does not increase 24-h fat oxidation. Exerc. Sport Sci. Rev. 37, 93–101. 10.1097/JES.0b013e31819c2f0b 19305201 PMC2885974

[B43] Melanson E. L. Sharp T. A. Seagle H. M. Horton T. J. Donahoo W. T. Grunwald G. K. (2002). Effect of exercise intensity on 24-h energy expenditure and nutrient oxidation. J. Appl. Physiol. 92, 1045–1052. 10.1152/japplphysiol.00706.2001 11842038

[B44] Netzer N. C. Chytra R. Kupper T. (2008). Low intense physical exercise in normobaric hypoxia leads to more weight loss in obese people than low intense physical exercise in normobaric sham hypoxia. Sleep Breath. 12, 129–134. 10.1007/s11325-007-0149-3 18057976 PMC2276561

[B45] Peronnet F. Massicotte D. (1991). Table of nonprotein respiratory quotient: an update. Can. J. Sport Sci. 16, 23–29. 1645211

[B46] Peronnet F. Massicotte D. Folch N. Melin B. Koulmann N. Jimenez C. (2006). Substrate utilization during prolonged exercise with ingestion of 13C-glucose in acute hypobaric hypoxia (4,300 m). Eur. J. Appl. Physiol. 97, 527–534. 10.1007/s00421-006-0164-2 16775741

[B47] Rapoport B. I. (2010). Metabolic factors limiting performance in marathon runners. PLoS Comput. Biol. 6:e1000960. 10.1371/journal.pcbi.1000960 20975938 PMC2958805

[B48] Roberts A. C. Butterfield G. E. Cymerman A. Reeves J. T. Wolfel E. E. Brooks G. A. (1996). Acclimatization to 4,300-m altitude decreases reliance on fat as a substrate. J. Appl. Physiol. (1985). 81, 1762–1771. 8904597 10.1152/jappl.1996.81.4.1762

[B49] Rossiter H. B. Kowalchuk J. M. Whipp B. J. (2006). A test to establish maximum O_2_ uptake despite no plateau in the O_2_ uptake response to ramp incremental exercise. J. Appl. Physiol. (1985). 100, 764–770. 10.1152/japplphysiol.00932.2005 16282428

[B50] Saunders P. U. Pyne D. B. Gore C. J. (2009). Endurance training at altitude. High Alt. Med. Biol. 10, 135–148. 10.1089/ham.2008.1092 19519223

[B51] Simonson D. C. DeFronzo R. A. (1990). Indirect calorimetry: methodological and interpretative problems. Am. J. Physiol. 258(3 Pt 1), E399–E412. 2180312 10.1152/ajpendo.1990.258.3.E399

[B52] Sutton J. R. Reeves J. T. Wagner P. D. Groves B. M. Cymerman A. Malconian M. K. (1988). Operation Everest II: oxygen transport during exercise at extreme simulated altitude. J. Appl. Physiol. (1985). 64, 1309–1321. 3132445 10.1152/jappl.1988.64.4.1309

[B53] Trombold J. R. Christmas K. M. Machin D. R. Kim I. Y. Coyle E. F. (2013). Acute high-intensity endurance exercise is more effective than moderate-intensity exercise for attenuation of postprandial triglyceride elevation. J. Appl. Physiol. (1985). 114, 792–800. 10.1152/japplphysiol.01028.2012 23372145

[B54] Voss J. D. Masuoka P. Webber B. J. Scher A. I. Atkinson R. L. (2013). Association of elevation, urbanization and ambient temperature with obesity prevalence in the United States. Int. J. Obes. (Lond). 37, 1407–1412. 10.1038/ijo.2013.5 23357956

[B55] Wagner P. D. Gale G. E. Moon R. E. Torre-Bueno J. R. Stolp B. W. Saltzman H. A. (1986). Pulmonary gas exchange in humans exercising at sea level and simulated altitude. J. Appl. Physiol. (1985). 61, 260–270. 3090012 10.1152/jappl.1986.61.1.260

[B56] Wiesner S. Haufe S. Engeli S. Mutschler H. Haas U. Luft F. C.. (2010). Influences of normobaric hypoxia training on physical fitness and metabolic risk markers in overweight to obese subjects. Obesity (Silver. Spring). 18, 116–120. 10.1038/oby.2009.193 19543214

[B57] Workman C. Basset F. A. (2012). Post-metabolic response to passive normobaric hypoxic exposure in sedendary overweight males: a pilot study. Nutr. Metab. (Lond). 9:103. 10.1186/1743-7075-9-103 23157699 PMC3546003

